# Effect of physical activity levels on oncological breast surgery recovery: a prospective cohort study

**DOI:** 10.1038/s41598-021-89908-8

**Published:** 2021-05-17

**Authors:** Ifat Klein, Leonid Kalichman, Noy Chen, Sergio Susmallian

**Affiliations:** 1 Department of Physical Therapy, Assuta Medical Center, Tel Aviv, Israel; 2 Department of Physical Therapy, Recanati School for Community Health Professions, Faculty of Health Sciences, Ben-Gurion University of the Negev, Beer Sheva, Israel; 3 Department of Surgery, Assuta Medical Center, 20 Habarzel Street, 69710 Tel Aviv, Israel; 4 Faculty of Medicine, Ben Gurion University of the Negev, Beer Sheva, Israel

**Keywords:** Cancer, Health care

## Abstract

After breast cancer (BC) surgery, women may experience a physical decline. The effect of physical activity (PA) on the course of recovery after BC surgery has not yet been thoroughly examined. To analyze the impact of physical activity performed by women undergoing breast cancer surgery on measures of function, range of motion, and self-efficacy. A prospective study was carried out in 157 patients who underwent surgery for BC between October 2018 and April 2019, divided into four groups according to the intensity of PA with 6 months follow-up. 50 sedentary patients and 107 active patients were enrolled; the mean age was 52.6. Women who performed physical activity, moderate to vigorous, demonstrated lower function disabilities (QuickDASH 2.22) compared with inactivity or light physical activity (QuickDASH 7.0, *p* < 0.001), with better shoulder flexion (159.0° vs. 150.7°, *p* = 0.007) and abduction (159.5° vs. 152.2°, *p* = 0.008). Higher PA levels, displayed in higher self-efficacy reports (9.5 vs. 8.8, *p* = 0.002), and return to prior job status (0.005). The PA level does not influence pain at one, three and 6 months postoperatively (*p* = 0.278, *p* = 0.304 and *p* = 0.304 respectively). High PA levels increase the risk of axillary web syndrome (*p* = 0.041), although, it reduces the incidence of chronic pain (*p* = 0.007). Women who practice physical activity recover better from BC surgery than sedentary women. The higher the intensity and frequency of training, the better the results. Vigorous activity cause axillary web syndrome, despite, it has a beneficial effect on lowering the rate of chronic pain.

## Introduction

Evidence for the health benefits of different sport disciplines was either inconclusive or tenuous^1^. While research has traditionally focused on the association between cardio-respiratory fitness and health outcomes, the association between muscular fitness and health status has recently received increased attention^2^.

The effects of physical or sport activity, frequency, and intensity in the recovery process of patients that underwent breast cancer (BC) surgery have not been analysed in depth.

In recent decades there was progress in prevention, diagnosis, and treatment of women with BC^3–6^. Nonetheless, surgery remains today one of the pillars in the treatment of breast carcinoma that has implications for postoperative quality of life (QOL).

Pain is the most frequent impairment after BC treatment with a strong relationship to perceived disability reported by 60% of the patients^7^. Additional impairments include reduction of grip-strength in 40% and restrictions of arm and shoulder movement in 35%, all of which have a negative impact on patients' QOL^8–10^. Physical activity (PA) has been found to be beneficial for women in improving postoperative function and reducing the restriction of movement^11^. Furthermore, exercise reduces the side effects of oncologic treatments including fatigue, depression, anxiety, sleep disturbances, and nausea associated with disease treatments^12^. Moreover, PA has been found to reduce the risk of recurrence of the disease, and a higher level of physical fitness was found to reduce the risk of mortality rates^13,14^.

Earlier studies examining the effect of regular exercise and the level of activity have been found to improves range of motion (ROM)^15^, reduces the length of hospitalization and sick leave after surgery^16^. While, women engaging in exercise preoperatively had greater shoulder flexion ROM at 1 month, which persisted over a year^17^. Furthermore, a higher preoperative level of PA is associated with a faster recovery three weeks post BC surgery^18^. Patients that did not exercise preoperatively are at higher risks for developing more severe pain at 1 and 3 months after surgery^19^. Moderate to vigorous PA may mitigate C-reactive protein levels that in the low inflammatory response is associated with morbidity and mortality in BC patients^20^. In contrast, vigorous exercise during the end of treatment can cause concerns specifically in regards to anemia, and weight loss^21^.

The purpose of this study was to examine the effect of different levels of preoperative and postoperative PA on shoulder function, flexion and abduction ROM, and self-efficacy in women after BC surgery.

## Material and methods

A prospective trial was conducted in a single medical center in women that underwent BC surgery between October 2018 and April 2019. The trial was approved by the Helsinki Assuta Medical Center Review Board (approval number: 0122-17 ASMC) and was registered on the National Institutes of Health's website (ClinicalTrials.gov; study identifier NCT03389204). All patients provided written informed consent before enrollment. All methods were carried out in accordance with relevant guidelines and regulations.

### Sample

The study includes 188 women that underwent BC surgery. Inclusion criteria included women 18 years of age and older with functional independence, a diagnosis of BC, and planned surgical intervention. Exclusion criteria for participation were: Benign disease of the breast, cognitive disorders, fibromyalgia or chronic pain disorders, neurological disorders causing permanent disability, previous breast surgery, lymphedema before the surgery, previous shoulder surgery or injuries causing limited ROM, severe systemic diseases.

### Study procedures

Subjects were evaluated using questionnaire for their activity levels and outcome measures before surgery and 1 month, 3 months, and 6 months after surgery. PA levels were recorded, using a self-reported adapted questionnaire, developed for the study. Participants were asked whether they exercise regularly, about the intensity of the activities (inactive, light, moderate, vigorous), and how many hours on average are they training, 1–2 per week, 3–5 per week, more than 5 h per week. The questionnaire is partially based on the international PA questionnaire^22^ and metabolic rate intensities (METs) that evaluate physical activities intensity^23^. Light PA (METs 1.1-3.0) included casual walking, stretching, Feldenkrais, Tai chi. Moderate PA (METs 3.0-6.0) included Pilates, Yoga, jogging, aerobics, bicycling, etc. Rigorous PA (METs > 6.0) included running, high-intensity fitness training, heavy lifting, swimming, and competitive sport such as basketball and football^24^.

### Outcome measures

The QuickDASH score^25^ was used for assessing the physical function and upper limbs activity. ROM (abduction and flexion) were evaluated using DrGoniometer application^26^. Self-efficacy which represents how much the participant felt that she returned to preoperative function on a scale of 1 (unable to carry out activities by themselves) to 10 (able to perform all activities by themselves). Additional measures included the number of sick days and if returned to the same job status. Pain levels were measured in each follow-up, using the numeric pain rating scale (NPRS), in values ranging 0–10^27^. Number of sick days and if the patients returned to prior job status.

### Statistical analysis

To calculate sample size estimation the PS Power and Sample Size Calculations software **(**Version 3.0, January 2009) was used. QuickDASH instrument was the main outcome measure used to evaluated shoulder functional disability. QuickDASH score^28^ after BC surgery (43.2 ± 18) was compared with the general population^29^ (10.1 ± 14.6) to estimate the sample size. Using this data and the probability of type I error was 0.05, and the probability of type II error was 0.2, 30 experimental subjects, and 30 control subjects were needed to reject the null hypothesis. Since 4 different groups were evaluated, with a probability of up to 20% dropping out of the study, 160 patients were planned to enter the study.

Statistical analysis was performed using the SPSS statistical package, Version 21 (SPSS Inc, Chicago, IL, USA). The significance level was set at *p* < 0.05.

The non-parametric Mann–Whitney rank-sum test for independent samples was applied for testing the statistical significance of the difference between continuous parameters. Nominal variables were evaluated by the chi-squared test. A repeated measures approach (mixed ANOVA) was used for analyzing study groups as well as group by time interactions.

## Results

**Figure 1:**
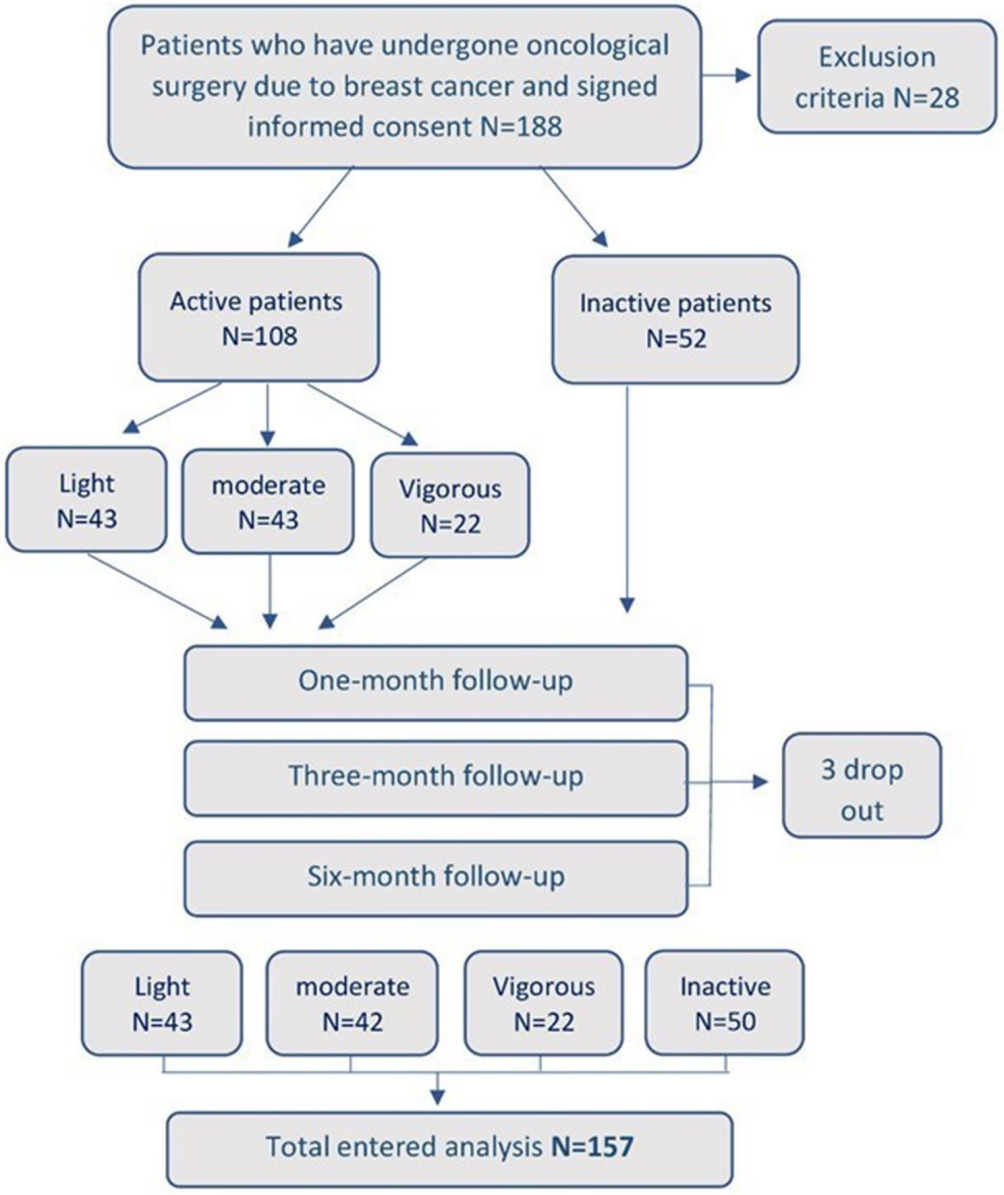
Study flow chart.

A total of 157 patients were eligible for inclusion (Fig. 1). Twenty-eight patients (14.89%) were excluded: one (3.6%) had a non-malignant fibroadenoma, 17 women (60.7%) had previous breast surgery, five (17.9%) had limited shoulder ROM mostly rotator cuff tears, two (7.1%) had lymphedema and three (11%) had fibromyalgia. Three patients were dropped out of the study due to incomplete data.

The study sample included 50 (31.8%) sedentary and 107 subjects that routinely exercise. The level of PA was divided according to the MET into light 43 (27.4%), moderate 42 (26.8%), and vigorous 22 (14.0%).

The mean patients' age was 52.2 and the mean BMI was 25.0 kg/m^2^. Most patients 77 (47.1%) had low stage cancer 1A, and the most common surgical procedure was a lumpectomy and sentinel lymph node biopsy 89 (56.7%), while 54 (34.4%) of patients had breast reconstruction procedures (Table 1). Patients in the inactive group received on average more neoadjuvant and radiation treatments compared with the active group (*p* = 0.023 and *p* < 0.001, respectively). Six months postoperative, mastectomy surgeries resulted in more functional limitation (QuickDASH 7.2 ± 11.1), pain (NPRS 1.1 ± 1.2) and decreased shoulder flexion (147.9° ± 22.5°) compared with lumpectomies (QuickDASH 3.6 ± 6.2, NPRS 0.5 ± 0.9 and 156.7° ± 12.6°), *p* = 0.022, *p* = 0.002 and *p* = 0.026 respectively.

**Table 1:** Baseline characteristics of the study population.

	Inactive N = 50	Active N = 107	*P* value
Age (yrs.)	53.8 ± 13.8	51.4 ± 12.4	0.321
BMI (kg/m^2^)	25.1 ± 4.1	24.9 ± 4.5	0.396
Dominance (RT)	42 (84.0%)	94 (87.9%)	0.335
Smokers	11 (22.0%)	13 (12.1%)	0.089
Hospital stay (days)	1.6 ± 0.8	1.5 ± 0.7	0.850
**Surgical treatments**			
Mastectomy	16 (32.0%)	39 (36.4%)	0.360
Lumpectomy	34 (68.0%)	68 (63.6%)	
ALNB	7 (14.0%)	7 (6.5%)	0.164
SLNB	43 (86.0%)	97 (90.7%)	
Reconstruction	15 (30.0%)	40 (37.4%)	0.236
**Non-surgical treatments**			
Neoadjuvant. C	17 (34.0%)	18 (16.8%)	0.023*
Chemotherapy	24 (48.0%)	36 (33.6%)	0.061
Radiation therapy	40 (80.0%)	56 (52.3%)	< 0.001*
**Number of sick days**			
1–7	4 (8.0%)	12 (11.2%)	0.901
8–14	7 (14.0%)	13 (12.1%)	
15–21	13 (26.0)	26 (24.3%)	
22–30	6 (12.0%)	18 (16.8%)	
More than 30	16 (32.0%)	32 (29.9%)	
**Postoperative complications**			
Infection	5 (10.0%)	6 (5.6%)	0.317
Seroma	5 (10.0%)	6 (5.6%)	0.317
Revision surgery	3 (6.0%)	1 (1.0%)	0.061
AWS (6 MNTH)	2 (4.1%)	14 (13.1%)	0.081
Lymphedema (6 MNTH)	6 (12.2%)	6 (5.7%)	0.334

Continuous variables are presented as mean and standard deviation (SD) and categorical variables are presented as number and percentage. Significant *p* value*p ≤ 0.05.

YRS, Years; BMI, Body mass index; kg/m^2^, Mass in kilograms divided by the square of the body height in meters; RT, Right; Neoadjuvant. C, Neoadjuvant Chemotherapy; ALND, Axillar lymph node dissection; SLNB, Sentinel lymph node biopsy; BRCA1-2, Breast Cancer gene; NPRS, Numeric pain rating scale; AWS, Axillar web syndrome; MNTH, Month.

Axillar lymph node procedures resulted in more pain (NPRS 1.4 ± 1.1), functional decline (QuickDASH 9.5 ± 8.7) and decreased shoulder abduction (149.8° ± 19.3°) compared with sentinel lymph node biopsy (NPRS 0.7 ± 1.0, QuickDASH 4.5 ± 8.3 and 155.8° ± 17.0°) or no axillar involvement (NPRS 0.0 ± 0.0, QuickDASH 0.0 ± 0.0 and 171.0° ± 10.1°), *p* = 0.016, *p* = 0.002 and *p* = 0.007 respectively.

### The effect of physical activity level on functional disabilities

**Figure 2:**
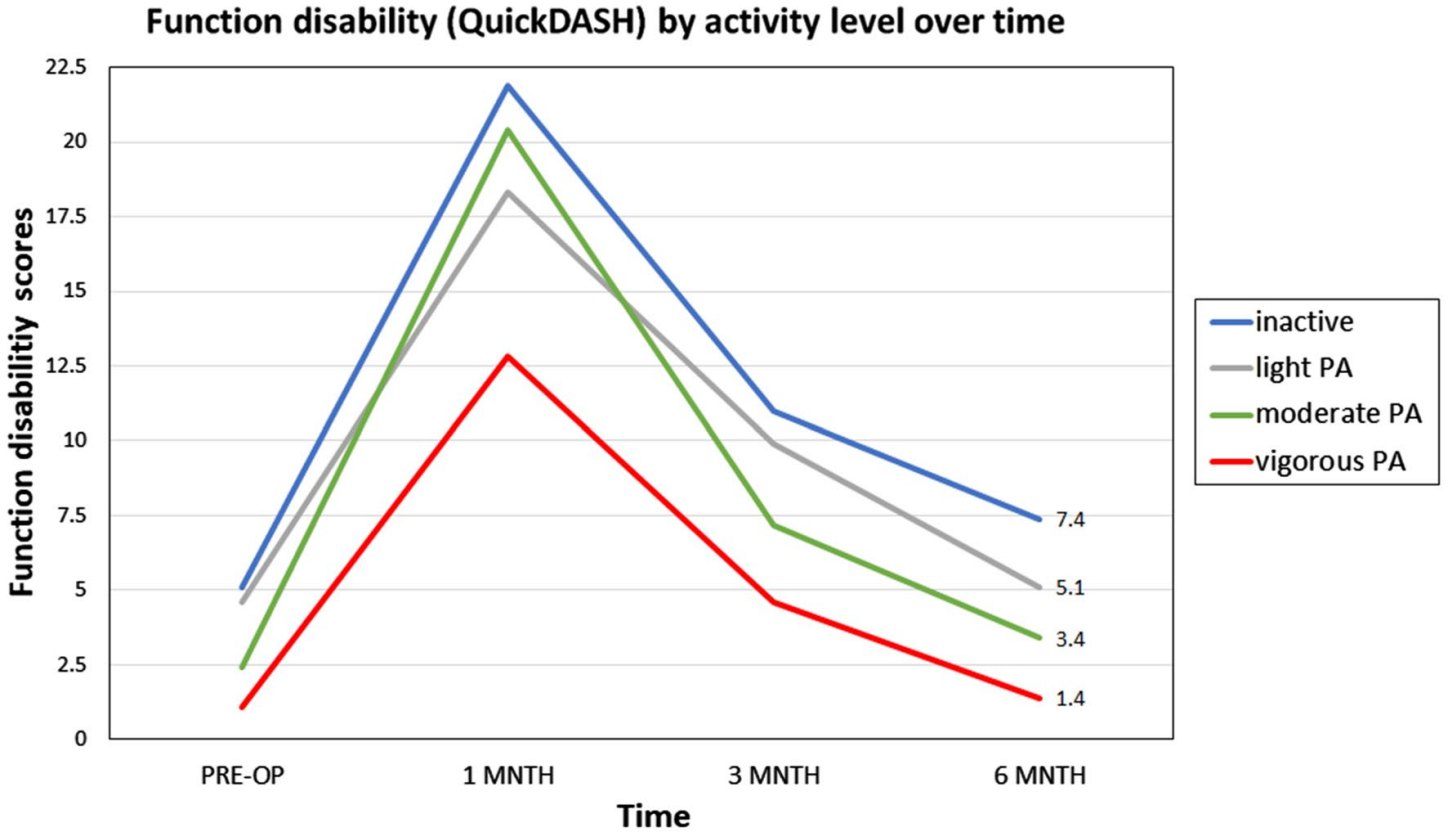
The effect of physical activity on function disability scores by activity level over time.

Throughout the study period, a higher preoperative intensity of the activity was associated with higher postoperative functional improvement. Three months postoperatively, patients performing vigorous PA before surgery, had less function disabilities (QuickDASH 4.6) compared to moderate PA (QuickDASH 7.2), light PA (QuickDASH 9.9), and inactive patients, (QuickDASH 11.0), *p* = 0.025, reported in Table 2. The trend was maintained 6 months postoperatively, *p* = 0.020. No significant differences were found regarding the influence of routine PA on function in the first month postoperative (Fig. 2).

**Table 2:** The effect of the physical activity intensity on function, ROM, pain, self-efficacy, job status and sick days classified by intensity levels.

Variable	Inactive N = 50	Light PA n = 43	Moderate n = 42	Vigorous n = 22	*p* value
**Function disabilities by (QuickDASH)**					
1 month	21.9 ± 17.3	18.3 ± 15.8	20.4 ± 18.3	12.8 ± 15.7	0.138
3 months	11.0 ± 14.0	9.9 ± 12.0	7.2 ± 7.6	4.6 ± 9.6	0.025*
6 months	7.4 ± 10.8	5.1 ± 8.6	3.4 ± 5.2	1.4 ± 3.7	0.020*
**ABD ROM**					
1 month	134.8 ± 27.0	143.8 ± 20.5	152.1 ± 19.3	154.5 ± 17.1	< 0.001*
3 months	146.0 ± 25.9	147.0 ± 18.9	158.8 ± 12.1	161.1 ± 11.7	< 0.001*
6 months	150.9 ± 18.2	151.4 ± 22.1	161.9 ± 9.0	161.2 ± 11.2	0.007*
**FLEX ROM**					
1 month	132.9 ± 33.0	144.8 ± 22.4	152.2 ± 19.1	151.2 ± 14.5	0.006*
3 months	144.3 ± 27.1	149.0 ± 19.6	155.6 ± 14.5	154.9 ± 13.4	0.079
6 months	150.7 ± 17.7	149.4 ± 18.3	157.7 ± 17.4	159.7 ± 9.8	0.010*
**Pain by (NPRS)**					
1 month	1.9 ± 1.3	1.7 ± 1.2	1.9 ± 1.4	1.3 ± 1.3	0.278
3 months	1.3 ± 1.1	1.3 ± 1.1	1.2 ± 1.2	0.8 ± 1.0	0.304
6 months	1.0 ± 1.1	0.6 ± 1.1	0.7 ± 1.0	0.6 ± 0.8	0.304
**Self-efficacy**					
1 month	7.7 ± 1.6	8.0 ± 1.8	7.5 ± 1.9	8.4 ± 1.8	0.133
3 months	8.5 ± 1.5	8.6 ± 1.3	9.0 ± 1.1	9.1 ± 1.1	0.153
6 months	8.7 ± 1.3	9.3 ± 1.0	9.3 ± 0.9	9.7 ± 0.5	0.005*
**Return to prior Job status**					
No	17 (34.0%)	14 (32.6%)	7 (16.7%)	5 (22.7%)	0.229
Yes	33 (66.0%)	29 (67.4%)	35 (83.3%)	17 (77.3)	
**Number of sick days**					
1–7	4 (8.7%)	4 (10.0%)	6 (15.0%)	2 (9.5%)	0.467
8–14	7 (15.2%)	3 (7.5%)	3 (7.5%)	7 (33.3%)	
15–21	13 (28.3%)	11 (27.5%)	11 (27.5%)	4 (19.0%)	
22–30	6 (13.0%)	9 (22.5%)	7 (17.5%)	2 (9.5%)	
More than 30	16 (34.8%)	13 (32.5%)	13 (32.5%)	6 (28.6%)	

Continuous variables are presented as mean and standard deviation (SD) and categorical variables are presented as number and percentage. Significant *p* value*p ≤ 0.05.

PA, Physical activity; ABD, Abduction; FLEX, Flexion; ROM, Range of motion; NPRS, Numeric pain rating scale.

### Flexion and abduction ROM

**Figure 3:**
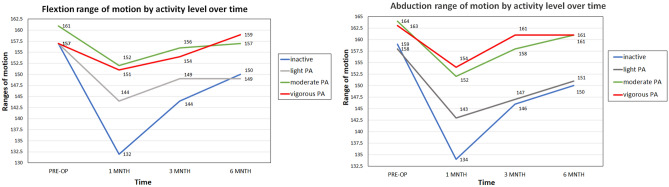
The effect of physical activity on abduction flexion range of motion by activity level over time.

The different preoperative exercise levels had a significant effect on the course of recovery in the values of shoulder ROM. Women who did not exercise measured lower ROM throughout the three follow-ups. After 1-month, inactive patients reported lower abduction ranges (134.8°) compared with light (143.8°), moderate (152.1°), and vigorous activity (154.5°), at the significance level of *p* < 0.001. After 6 months, the differences between the groups narrowed and remained significant *p* = 0.007 (Fig. 3).

The shoulder flexion ROM improved in the same trend, so women who exercised preoperatively improved their ROM largely in the months after surgery compared with inactive patients. The moderate and vigorous activity levels had similar effect (152.2° and 151.2°), with the main differences being between the moderate activity (144.8°) and inactive patients (132.9°), at significance level of *p* = 0.006. Similar differences were found after 6-months, *p* = 0.010.

### Pain and self-efficacy scores

Pain was not found to be affected by the level of women's PA throughout the study at 1, 3, and 6 months postoperatively (*p* = 0.278, *p* = 0.304, *p* = 0.304, respectively), reported in Table 2.

Self-efficacy, improved as expected over time. Even though, the level of preoperative PA was found to affect self-efficacy only at 6-months follow-up with a significance level of *p* = 0.005.

### The influence of intensity of physical activity

To examine separately the effect of training intensity on outcome measures, vigorous and moderate PA were compared with light PA or inactivity. Six months after surgery, women who performed moderate to high-intensity PA reported less functional limitation (QuickDASH 2.2 ± 4.2) compared to women who did not exercise or performed light PA (QuickDASH 7.0 ± 10.2) with a significance of *p* < 0.001. The abduction ranges were better (159.5° ± 13.4°) relative to the light activity or inactivity (152.2° ± 19.4°), at significance level *p* = 0.008. Furthermore, the flexion ROM was higher of active patients (157° ± 17.9°) compared to low activity (150.7° ± 15.9°), significance level *p* = 0.007. In addition, self-efficacy was higher in active women at higher intensity and pain levels were lower, relative to women in the light or inactive group (*p* = 0.002 and *p* = 0.014, respectively). Moreover, the more active women also returned to the same job status in higher percentages (*p* = 0.005).

### The influence of frequency of physical activity

The frequency of PA had a positive effect on patients' recovery. Six months postoperatively, sedentary women reported greater shoulder functional limitations, resulting in QuickDASH 8.5 ± 11.8. Training 1–2 h per week reported QuickDASH 4.4 ± 6.4; those who trained 3–5 h per week had QuickDASH of 2.1 ± 4.3. No functional limitations were reported by women who exercised more than 5 h per week resulting in QuickDASH 0 (*p* = 0.002), reported in Table 3.

**Table 3:** The influence of frequency of physical activities.

Variable	Duration of PA (weekly training hours)				
	0	1–2	3–5	< 5	*p* value
QuickDASH	8.5 ± 11.8	4.4 ± 6.4	2.1 ± 4.3	0 ± 0	0.002*
ABD ROM	151.1 ± 22.0	151.9 ± 16.4	161.9 ± 10.5	164.0 ± 6.3	0.002*
FLEX ROM	150.2 ± 20.4	148.0 ± 18.9	160.7 ± 10.5	162.0 ± 7.1	< 0.001*
Self- efficacy	8.4 ± 1.3	9.2 ± 0.8	9.6 ± 0.5	10 ± 0	< 0.001*
Pain	1.0 ± 1.1	0.7 ± 1 s.2	0.6 ± 0.9	0.6 ± 0.8	0.323
**Return to Job**					
No	24 (47.1%)	11 (23.4%)	7 (12.7%)	0	
Yes	27 (52.9%)	36 (76.6%)	48 (87.3%)	4 (100%)	< 0.001*
**Sick days**					
1–7	5 (10.6%)	3 (6.7%)	7 (13.5%)	0	
8–14	9 (17.6%)	1 (2.2%)	10 (19.2%)	0	0.047*
15–21	12 (25.5%)	18 (40.0%)	8 (15.4%)	2 (50.0%)	
22–30	3 (5.9%)	8 (17.8%)	13 (25.0%)	1 (25.0%)	
over 30	18 (38.3%)	15 (33.3%)	14 (26.9%)	1 (25.0%)	

Continuous variables are presented as mean and standard deviation (SD) and categorical variables are presented as number and percentage. Significant *p* value*p ≤ 0.05.

PA, Physical activity; ABD, Abduction; FLEX, Flexion; ROM, Range of motion; NPRS, Numeric pain rating scale.

Similarly, the higher the frequency of training, the higher the ROM of flexion and abduction (*p* = 0.002 and *p* < 0.001, respectively). Furthermore, self-efficacy was higher for women who had frequently trained (*p* < 0.001). Moreover, it was found that the higher the frequency of PA, the need for sick days decreased (*p* = 0.047) and the percentage of patients return to the preoperative job increased (*p* < 0.001). The frequency of activity did not affect pain values and number of sick days.

### Physical activity trends during the study period

Comparing between the women who were inactive and active throughout the study period (regardless of the type of activity), it was found that 6 months postoperatively, women in the PA group recovered better in measures of function QuickDASH 4.4 ± 2.9 compared with inactive patients QuickDASH 9.3 ± 12.5 (*p* = 0.006). Their abduction and flexion ROM were better 158.6° ± 14.1° and 155.4° ± 16.2° compared with inactive patients 149.5° ± 20.0° and 148.8° ± 20.5° (*p* = 0.007 and *p* = 0.044 respectively). Similar results were obtained for self-efficacy, active 10.4 ± 8.6 compared with inactive 8.6 ± 1.4 (*p* < 0.001) and even returned in higher percentages to the same work status (*p* < 0.001).

### Adverse effects of physical activity

No effect on exercise intensity was found on the incidence of seroma, infections, and lymphedema. However, high PA levels increase the risk of axillary web syndrome (*p* = 0.041). On the other hand, the higher the intensity of training, the risk of chronic pain (lasting more than 3 months) decreases significantly (*p* = 0.007), reported in Table 4.

**Table 4:** Postoperative adverse effects.

Variable	Inactive N = 50	Active N = 105			*p* value
Six months		Light n = 36	Moderate n = 49	Vigorous n = 20	
Seroma	4 (7.8%)	3 (8.3%)	3 (6.0%)	1 (5.0%)	0.950
Infection	4 (7.8%)	3 (8.3%)	4 (8.0%)	4 (8.0%)	0.632
Lymphedema	5 (12.5%)	3 (8.3%)	7 (14.0%)	0 (0%)	0.336
AWS	2 (4.1%)	2 (5.6%)	10 (20.4%)	2 (10.0%)	0.041*
Chronic pain	1.1 ± 1.3	0.6 ± 0.9	0.8 ± 1.0	0.2 ± 0.5	0.007*

Continuous variables are presented as mean and standard deviation (SD) and categorical variables are presented as number and percentage. Significant *p* value (< 0.05).

AWS, axillar web syndrome.

## Discussion

Preoperative and pre-diagnosis sport activity had a major positive effect on the recovery process of oncologic BC surgery^30^. Following what was reported by Nilsson that a higher intensity of the preoperative PA level is associated with a faster self-reported physical recovery^17^, in this cohort preoperative PA improved functional disabilities, abduction and flexion ROM during the 6 months postoperatively, compared to sedentary women. In addition, the higher the intensity of the exercise, the better the recovery.

Sedentary patients needed more radiotherapy and neoadjuvant treatments, which may indicate that physically active patients have an earlier diagnosis of the disease or a less aggressive presentation, which is an important point for future research. Physical activity can reduce cancer recurrence, mortality^31^ and improve health outcomes of BC survivors^32^. Additionally, among tamoxifen treated BC survivors, high PA levels before and after diagnosis were found to improve overall survival compared with inactive patients^33^.

Postoperative exercises for BC patients are effective in reducing pain and improving ROM^11,34–36^. The results from this study are a continuation of previous publications and attempt to give a broader picture of the effect of exercise on the course of recovery. Six months postoperatively, a return to exercise contributed to an improvement in function limitations and ROM. Unlike previous findings^11^, it was not found that PA had an effect on pain reduction. This finding can be explained because, during hospitalization, patients were instructed to report any type of pain and the to receive apropiate treatment^37^. Furthermore, the patients received instructions on how to cote with pain during daily activities and physical affair. Both can contribute to pain reduction, due to the emphasis in pain management.

Pickett et al. reported that women who exercised regularly before receiving a BC diagnosis, attempted to maintain their exercise programs, and therefore BC survivors who lead sedentary lifestyles are the ones that may benefit more from a structured exercise program^38^. In our results, women who were sedentary before surgery and started PA postoperatively did not improve their function, ROM and pain levels, or self-efficacy significantly, compared to sedentary women, although this may be attributed to the small sample of patients given for comparison, further studies may be necessary to corroborate this issue.

The effect of high intensity PA evaluated during chemotherapy, showed that vigorous training was more beneficial than moderate or low PA in preventing side effects of treatment in terms of QOL, strength, fatigue, body weight and return to routine activities^39,40^. Our results are consistent, finding that the higher the intensity of training, the greater the effect on recovery from surgery.

Another aspect is the effect of the duration of PA rather than intensity, as previous study found that higher duration of PA was associated with better improvement in shoulder ROM^41^. Our results demonstrated that the longer duration of PA was associated with fewer functional limitations, higher self-efficacy, better flexion, and abduction ROM, and the need for less sick days.

Moreover, regular PA helped patients to return to previous job status compared with inactive women. Lee et al. have reported similar results, finding patients who exercise before, during, and after treatment for BC are more likely to return to prior work^42^.

The results of the study did not show associations between the level of PA and the degree of acute postoperative short-term complications such as seroma, bleeding, infections and nor the long-term complication of lymphedema. Until recently, intense PA has been associated with the development of lymphedema after BC^43^. New studies have examined the effect of intense training including weight lifting after breast surgery and have found no direct link to the development or exacerbation of lymphedema^44,45^.

Axillar web syndrome (AWS) is a self-limiting complication of axillar surgery^46^. In this study, we found an association between vigorous PA performance and risk of developing AWS. The authors who previously examined risk factors for developing AWS found no relationship between training level and increased risk of AWS^47,48^, with the exception of a case report that described AWS development after physical effort^49^. Moreover, among the risk factors for the development of AWS should be considered younger age, greater time elapsed since surgery and number of resected lymph nodes^50^, but PA was not considered.

Chronic pain in BC surgery appears after 3 months, the risk factors being extension of the surgery, intensity of acute pain and radiotherapy^51^. A positive association was found between the level of intensity of the PA and of chronic pain, the higher the activity levels the lower the incidence of chronic pain, and the severity of the pain. These results are a continuation of the results of Forsythe et al. who reported that weight gain and lack of PA place BC survivors at risk for pain long after treatment ends^52^. In addition, Galiano-Castillo et al. found that performing tailored online training for 8 weeks improved pain severity, compared with control^53^.

The limitations of the study are based on the complexity and possible variations of the pre- and post-operative treatments. Long-term follow-up is necessary to verify the effect of sport on chronic disabilities.

The strength of the study was in a broad examination of the effect of exercise before and after surgery on the course of recovery that allows us to affirm that sport is beneficial in the postoperative recovery and can be practiced without fear of causing complications. Furthermore, the separation into groups of patients according to intensity and frequency of PA provides relevant data from the present study.

## Conclusion

Physical activity improves recovery in patients who underwent surgery for breast cancer. Physical activity resulted in improved function, range of motion, self-efficacy, pain, with higher return to prior work. The higher the intensity and frequency of training, the greater the was the improvement. Vigorous exercise may cause more cases of axillar web syndrome, however also reduce the incidence of prolonged postoperative pain.

## Supplementary Information

Supplementary Table 1.

Supplementary Table 2.

Supplementary Table 3.

Supplementary Table 4.

## Abbreviations

ABD: Abduction
ALND: Axillary lymph node dissection
BMI: Body mass index
BC: Breast cancer
FLEX: Flexion
NPRS: Numeric pain rating scale
PA: Physical activity
QOL: Quality of life
ROM: Range of movement
°: Degrees

## References

[1] Oja P, Titze S, Kokko S (2015). Health benefits of different sport disciplines for adults: Systematic review of observational and intervention studies with meta-analysis. Br J Sports Med.

[2] Smith JJ, Eather N, Morgan PJ (2014). The health benefits of muscular fitness for children and adolescents: A systematic review and meta-analysis. Sports Med.

[3] Gradishar WJ, Bland KI, Klimberg VS (2017). The Breast: Comprehensive Management of Benign and Malignant Diseases.

[4] Schiff R, Chamness GC, Brown PH (2003). Advances in breast cancer treatment and prevention: Preclinical studies on aromatase inhibitors and new selective estrogen receptor modulators (SERMs). Breast Cancer Res..

[5] Costa RL, Czerniecki BJ (2020). Clinical development of immunotherapies for HER2 breast cancer: A review of HER2-directed monoclonal antibodies and beyond. NPJ Breast Cancer..

[6] Ontario HQ (2020). Gene expression profiling tests for early-stage invasive breast cancer: A health technology assessment. Ont Health Technol Assess Ser..

[7] Ilhan E, Chee E, Hush J (2017). The prevalence of neuropathic pain is high after treatment for breast cancer: a systematic review. Pain.

[8] Bodai BI, Tuso P (2015). Breast cancer survivorship: A comprehensive review of long-term medical issues and lifestyle recommendations. Perm J..

[9] Rietman DP, Debreczeni R (2004). Impairments, disabilities and health related quality of life after treatment for breast cancer: A follow-up study 27 years after surgery. Disability Rehabil..

[10] Nesvold I, Fosså SD, Holm I (2010). Arm/shoulder problems in breast cancer survivors are associated with reduced health and poorer physical quality of life. Acta Oncol..

[11] De Groef A, Van Kampen M, Dieltjens E (2015). Effectiveness of postoperative physical therapy for upper limb impairments following breast cancer treatment: A systematic review. Arch Phys Med Rehabil..

[12] Mustian KM, Sprod LK, Palesh OG (2009). Exercise for the management of side effects and quality of life among cancer survivors. Curr Sports Med Rep..

[13] McNeely M, Campbell K, Rowe B (2006). Effects of exercise on breast cancer patients and survivors: A systematic review and meta-analysis. CMAJ.

[14] Volaklis KA, Halle M, Tokmakidis SP (2013). Exercise in the prevention and rehabilitation of breast cancer. Wien Klin Wochenschr..

[15] Spei M, Samoli E, Bravi F (2019). Physical activity in breast cancer survivors: A systematic review and meta-analysis on overall and breast cancer survival. Breast.

[16] McNeely M, Campbell K, Ospina M (2010). Exercise interventions for upper-limb dysfunction due to breast cancer treatment. Cochrane Database Syst. Rev..

[17] Nilsson H, Angerås U, Bock D (2016). Is preoperative physical activity related to post-surgery recovery? A cohort study of patients with breast cancer. BMJ Open.

[18] Smoot B, Paul SM, Aouizerat BE (2016). Predictors of altered upper extremity function during the first year after breast cancer treatment. Am. J. Phys. Med. Rehabil..

[19] Baima J, Reynolds S, Edmiston K (2017). Teaching of independent exercises for prehabilitation in breast cancer. J Cancer Educ..

[20] Sabiston CM, Wrosch C, Castonguay AL (2018). Changes in physical activity behavior and C-reactive protein in breast cancer patients. Ann. Behav. Med..

[21] Speck RM, Courneya KS, Mâsse LC (2010). An update of controlled physical activity trials in cancer survivors: A systematic review and meta-analysis. J. Cancer Surv..

[22] Craig CL, Marshall AL, Sjöström M (2003). International physical activity questionnaire: 12-country reliability and validity. Med Sci Sports Exerc..

[23] Ainsworth BE, Haskell WL, Whitt MC (2000). Compendium of physical activities: An update of activity codes and MET intensities. Med. Sci. Sports Exerc..

[24] Stefani L, Galanti G, Klika R (2017). Clinical implementation of exercise guidelines for cancer patients: Adaptation of ACSM’s guidelines to the Italian model. J. Funct. Morphol. Kinesiol..

[25] Gummesson C, Ward MM, Atroshi I (2006). The shortened disabilities of the arm, shoulder and hand questionnaire (quick DASH): Validity and reliability based on responses within the full-length DASH. BMC Musculoskelet Disord..

[26] Vercelli S, Sartorio F, Bravini E (2017). DrGoniometer: A reliable smartphone app for joint angle measurement. Br. J. Sports Med..

[27] Williamson A, Hoggart B (2005). Pain: a review of three commonly used pain rating scales. J. Clin. Nurs..

[28] Harder H, Langridge C, Solis-Trapala I (2015). Post-operative exercises after breast cancer surgery: Results of a RCT evaluating standard care versus standard care plus additional yoga exercise. Eur. J. Integr. Med..

[29] Hunsaker F, Cioffi D, Amadio P (2002). The american academy of orthopaedic surgeons outcomes instruments : Normative values from the general population. J. Bone Joint Surg. Am..

[30] Yang A, Sokolof J, Gulati A (2018). The effect of preoperative exercise on upper extremity recovery following breast cancer surgery: A systematic review. Int. J. Rehabil. Res..

[31] Nelson SH, Marinac CR, Patterson RE, Nechuta SJ, Flatt SW, Caan BJ, Kwan ML, Poole EL, Chen WY, Shu X, Pierce JP (2016). Impact of very low physical activity, BMI, and comorbidities on mortality among breast cancer survivors. Breast Cancer Res. Treat..

[32] Bach PB, Schrag D, Brawley OW, Galaznic A, Yakren S, Begg CB (2002). Survival of blacks and whites after a cancer diagnosis. JAMA.

[33] De Glas NA (2014). Physical activity and survival of postmenopausal, hormone receptor–positive breast cancer patients: Results of the Tamoxifen Exemestane Adjuvant Multicenter Lifestyle study. Cancer.

[34] Ribeiro IL, Moreira RFC, Ferrari AV (2019). Effectiveness of early rehabilitation on range of motion, muscle strength and arm function after breast cancer surgery: A systematic review of randomized controlled trials. Clin Rehabil..

[35] Wilson DJ (2017). Exercise for the patient after breast cancer surgery. Semin Oncol. Nurs..

[36] Galantino ML, Stout NL (2013). Exercise interventions for upper limb dysfunction due to breast cancer treatment. Phys. Ther..

[37] Lanser P, Gessel S (2001). Pain management: The fifth vital sign. Healthc. Benchmarks.

[38] Pickett M, Mock V, Ropka ME (2002). Adherence to Moderate-Intensity exercise during breast cancer therapy. Cancer Pract..

[39] Mijwel S, Jervaeus A, Bolam KA (2019). High-intensity exercise during chemotherapy induces beneficial effects 12 months into breast cancer survivorship. J. Cancer Surv..

[40] Schulz SVW, Laszlo R, Otto S (2018). Feasibility and effects of a combined adjuvant high-intensity interval/strength training in breast cancer patients: A single-center pilot study. Disabil. Rehabil..

[41] Levy EW, Pfalzer LA, Danoff J (2012). Predictors of functional shoulder recovery at 1 and 12 months after breast cancer surgery. Breast Cancer Res. Treat..

[42] Lee MK, Kang HS, Lee KS (2017). Three-year prospective cohort study of factors associated with return to work after breast cancer diagnosis. J. Occup. Rehabil..

[43] Johansson K, Tibe K, Weibull A, Newton RU (2005). Low intensity resistance exercise for breast cancer patients with arm lymphedema with or without compression sleeve. Lymphology.

[44] Schmitz KH, Ahmed RL, Troxel A (2009). Weight lifting in women with breast-cancer–related lymphedema. N. Engl. J. Med..

[45] Ahmed RL, Thomas W, Yee D (2006). Randomized controlled trial of weight training and lymphedema in breast cancer survivors. J. Clin. Oncol..

[46] Aydogan F, Belli AK, Baghaki S, Karabulut K, Tahan G, Uras C (2008). Axillary web syndrome after sentinel node biopsy. Breast Care (Basel)..

[47] Fukushima KFP, Carmo LA, Borinelli AC (2015). Frequency and associated factors of axillary web syndrome in women who had undergone breast cancer surgery: A transversal and retrospective study. Springerplus.

[48] Jacob T, Bracha J (2019). Identification of signs and symptoms of axillary web syndrome and breast seroma during a course of physical therapy 7 months after lumpectomy: A case report. Phys Ther..

[49] Hunt WTN, Porter R, Lucke T (2020). Axillary web syndrome induced by physical exertion. Clin. Exp. Dermatol..

[50] Fukushima KF, Carmo LA, Borinelli AC, Ferreira CW (2015). Frequency and associated factors of axillary web syndrome in women who had undergone breast cancer surgery: A transversal and retrospective study. Springerplus.

[51] Poleshuck EL, Katz J, Andrus CH, Hogan LA, Jung BF, Kulick DI, Dworkin RH (2006). Risk factors for chronic pain following breast cancer surgery: A prospective study. J. Pain..

[52] Forsythe LP, Alfano CM, George SM (2013). Pain in long-term breast cancer survivors: The role of body mass index, physical activity, and sedentary behavior. Breast Cancer Res. Treat..

[53] Galiano-Castillo N, Cantarero-Villanueva I, Fernández-Lao C (2016). Telehealth system: A randomized controlled trial evaluating the impact of an internet-based exercise intervention on quality of life, pain, muscle strength, and fatigue in breast cancer survivors. Cancer.

