# Supplementary material for: Effect of physical activity levels on oncological breast surgery recovery: a prospective cohort study
Source: Sci Rep. 2021 May 17;11:10432. doi: 10.1038/s41598-021-89908-8 (PMC8129134; doi:10.1038/s41598-021-89908-8)
Supplement: Supplementary file 1 — Supplementary Table 1. [file 41598_2021_89908_MOESM1_ESM.docx]

**Table 5.** **The influence of intensity physical activity divided by active or inactive on outcome measures.**

| Variable | Inactive throughout the study | Active throughout the study | p- value |
| --- | --- | --- | --- |
| 6 months | N=27 | N=82 |  |
| QuickDASH  ABD ROM  FLEX ROM  Self- efficacy  Pain  Sick days  Until 7  8-14  15-21  22-30  More than 30  Return to Job  No  Yes | 9.3±12.5  149.5±20.0  148.8±20.5  8.6±1.4  1.2±0.9  3(10.7%)  6(21.4%)  6(21.4%)  2(7.1%)  11(39.3%)  12(44.4%)  15(55.6%) | 4.4±2.9  158.6±14.1  155.4±16.2  10.4±8.6  0.6±0.8  10(12.2%)  10(12.2%)  20(24.4%)  17(20.7%)  25(30.5%)  13(14.8%)  75(85.2%) | **0.006**  **0.007**  **0.044**  **0.000**  **0.001**  0.397  **0.001** |

Continuous variables are presented as mean and standard deviation (SD) and categorical variables are presented as number and percentage. Significant p-value*p***≤*** 0.05.

Abbreviations: PA: Physical activity, ABD: Abduction, FLEX: Flexion, ROM: Range of motion, pain using Numeric pain rating scale.
