# Supplementary material for: Effect of physical activity levels on oncological breast surgery recovery: a prospective cohort study
Source: Sci Rep. 2021 May 17;11:10432. doi: 10.1038/s41598-021-89908-8 (PMC8129134; doi:10.1038/s41598-021-89908-8)
Supplement: Supplementary file 2 — Supplementary Table 2. [file 41598_2021_89908_MOESM2_ESM.docx]

**Table 6.** **The influence of intensity physical activity divided by two activity groups on outcome measures.**

|  | Intensity of PA | | |
| --- | --- | --- | --- |
| Variable | Inactive+  light PA | Moderate+ Vigorous PA | p- value |
| Preoperative | N=81 | N=69 |  |
| QuickDASH  ABD ROM  FLEX ROM | 4.9±7.6  159.3±9.7  157.6±10.8 | 1.9±4.7  164.0±8.3  160.07.6 | **0.002**  **0.005**  0.226 |
| 6 months | N=81 | N=67 |  |
| QuickDASH  ABD ROM  FLEX ROM  Self- efficacy  Pain  Sick days  Until 7  8-14  15-21  22-30  More than 30  Return to Job  No  Yes | 7.0±10.2  152.2±19.4  150.7±17.9  8.8±1.3  1.0±1.1  7(8.6%)  11(13.6%)  20(24.7%)  12(14.8%)  31(38.3%)  31(35.6%)  56(54.4%) | 2.2±4.2  159.5±13.4  157.0±15.9  9.5±0.6  0.6±0.8  8(11.9%)  9(13.4%)  20(29.9%)  13(19.4%)  17(25.4%)  11(15.7%)  59(84.3%) | **0.001**  **0.008**  **0.007**  **0.002**  **0.014**  0.542  **0.005** |

Continuous variables are presented as mean and standard deviation (SD) and categorical variables are presented as number and percentage. Significant p-value*p***≤*** 0.05.

Abbreviations: PA: Physical activity, ABD: Abduction, FLEX: Flexion, ROM: Range of motion, pain using Numeric pain rating scale.
