# Supplementary material for: Effect of physical activity levels on oncological breast surgery recovery: a prospective cohort study
Source: Sci Rep. 2021 May 17;11:10432. doi: 10.1038/s41598-021-89908-8 (PMC8129134; doi:10.1038/s41598-021-89908-8)
Supplement: Supplementary file 3 — Supplementary Table 3. [file 41598_2021_89908_MOESM3_ESM.docx]

**Table 7**. **The influence of type of surgery on outcome measures.**

| Variable | Lumpectomy  N=55 | Mastectomy  n=102 | p-value | No axillar dissection  n=3 | SLNB  n=132 | ALND  n=12 | p-value |
| --- | --- | --- | --- | --- | --- | --- | --- |
| Function disabilities by (QuickDASH) | | | | | | | |
| 1 month  3 months  6 months | 14.9±15.4  7.0±10.7  3.6±6.2 | 26.7±17.4  12.1±12.4  7.2±11.1 | <0.001*  0.001*  0.022* | 1.5±2.5  1.5±2.5  0.0±0.0 | 19.1±16.9  8.6±11.6  4.5±8.3 | 24.7±17.6  11.8±11.4  9.5±8.7 | 0.035*  0.140  0.002* |
| ABD ROM | | | | | | | |
| 1 month  3 months  6 months | 148.3±20.2  152.6±18.5  157.9±13.4 | 138.7±26.9  150.3±22.9  151.1±22.2 | 0.043*  0.601  0.112 | 157.3±15.1  163.3±14.5  171.0±10.1 | 145.5±22.9  152.0±20.3  155.8±17.0 | 134.2±26.1  147.6±20.0  149.8±19.3 | <0.001*  <0.001*  0.007* |
| FLEX ROM | | | | | | | |
| 1 month  3 months  6 months | 147.8±24.6  151.6±19.2  156.7±12.6 | 137.3±26.4  147.5±23.3  147.9±22.5 | 0.007*  0.257  0.026* | 155.6±11.3  151.3±1.5  156.6±5.8 | 144.2±25.7  150.0±20.9  154.1±17.4 | 139.1±28.0  151.5±22.1  147.5±16.7 | 0.666  0.706  0.232 |
| Pain by (NPRS) | | | | | | | |
| 1 month  3 months  6 months | 1.5±1.2  1.0±1.1  0.5±0.9 | 2.3±1.4  1.6±1.2  1.1±1.2 | 0.001*  0.003*  0.002* | 0.0±0.0  0.0±0.0  0.0±0.0 | 1.8±1.3  1.2±1.1  0.7±1.0 | 2.4±1.3  1.6±1.0  1.4±1.1 | 0.023*  0.041*  0.016* |
| Self-efficacy | | | | | | | |
| 1 month  3 months  6 months | 8.3±1.5  9.0±1.2  9.3±0.8 | 6.9±1.8  8.3±1.5  8.9±1.4 | <0.001*  0.006*  0.255 | 10.0±0.0  10.0±0.0  10.0±0.0 | 7.8±1.8  8.7±1.3  9.2±1.0 | 7.5±1.2  8.5±1.1  8.5±0.9 | 0.027*  0.079  0.002* |

Continuous variables are presented as mean and standard deviation (SD) and categorical variables are presented as number and percentage. Significant p-value*p≤ 0.05.

Abbreviations: PA: Physical activity, ABD: Abduction, FLEX: Flexion, ROM: Range of motion, NPRS: Numeric pain rating scale.
