# Supplementary material for: Effect of physical activity levels on oncological breast surgery recovery: a prospective cohort study
Source: Sci Rep. 2021 May 17;11:10432. doi: 10.1038/s41598-021-89908-8 (PMC8129134; doi:10.1038/s41598-021-89908-8)
Supplement: Supplementary file 4 — Supplementary Table 4. [file 41598_2021_89908_MOESM4_ESM.docx]

**Table 8.** **The influence of type of breast reconstruction on outcome measures.**

| Variable | Breast reconstruction  N=55 | No breast reconstruction  n=102 | p-value |
| --- | --- | --- | --- |
| Function disabilities by (QuickDASH) | | | |
| 1 month  3 months  6 months | 25.7±17.2  11.5±12.1  6.6±10.6 | 15.5±15.9  7.3±10.9  3.9±6.8 | <0.001*  0.004*  0.069 |
| ABD ROM | | | |
| 1 month  3 months  6 months | 140.2±26.8  152.4±21.5  153.1±19.9 | 147.4±20.5  151.5±19.4  156.8±15.7 | 0.138  0.684  0.312 |
| FLEX ROM | | | |
| 1 month  3 months  6 months | 140.3±25.0  148.9±22.5  149.4±21.1 | 146.1±26.0  150.9±19.9  155.8±14.4 | 0.078  0.618  0.055 |
| Pain by (NPRS) | | | |
| 1 month  3 months  6 months | 2.3±1.4  1.6±1.2  1.1±1.2 | 1.5±1.2  1.0±1.1  0.5±0.9 | 0.001*  0.005*  0.004* |
| Self-efficacy | | | |
| 1 month  3 months  6 months | 6.9±1.9  8.4±1.5  8.9±1.3 | 8.5±1.5  8.9±1.2  9.3±0.8 | <0.001*  0.013*  0.207 |

Continuous variables are presented as mean and standard deviation (SD) and categorical variables are presented as number and percentage. Significant p-value*p≤ 0.05.

Abbreviations: PA: Physical activity, ABD: Abduction, FLEX: Flexion, ROM: Range of motion, NPRS: Numeric pain rating scale.
